# Towards ROXAS AI: automatic multi-species ring boundaries segmentation as regression in anatomical images

**DOI:** 10.3389/fpls.2025.1516635

**Published:** 2025-05-06

**Authors:** Marc Katzenmaier, Vivien Sainte Fare Garnot, Jan Dirk Wegner, Georg von Arx

**Affiliations:** ^1^ EcoVision Lab, Department of Mathematical Modeling and Machine Learning, University Zurich, Zurich, Switzerland; ^2^ Swiss Federal Institute for Forest, Snow and Landscape Research WSL, Birmensdorf, Switzerland; ^3^ Oeschger Centre for Climate Change Research, University of Bern, Bern, Switzerland

**Keywords:** tree ring, deep learning, quantitative wood anatomy, image segmentation, neural network, shrubs, ROXAS

## Abstract

**Introduction:**

Quantitative wood anatomy (QWA) along a time series of tree rings (known as tree-ring anatomy or dendroanatomy) has proven to be very valuable for reconstructing climate and for investigating the responses of trees and shrubs to environmental influences. A major obstacle to a wider use of QWA is the time- consuming data production, which also requires specialized equipment and expertise. This is why the research community has been striving to reduce these limitations by defining and improving tools and protocols along the entire data production chain. One of the remaining bottlenecks is the analysis of anatomical images, which broadly consists of cell and ring segmentation, followed by manual editing, measurements, and output. While dedicated software such as ROXAS can perform these tasks, its accuracy and efficiency are limited by its reliance on classical image analysis techniques. However, the reliability and accuracy of automatic cell and ring detection are key to efficient QWA data production.

**Methods:**

In this paper, we target automatic ring segmentation and deliberately focus on the most challenging case, circular ring structures in arctic angiosperm shrubs with partly very narrow and wedging rings. This shape requires high precision combined with a large global context, which is a challenging combination for instance segmentation approaches. We present a new iterative regression-based method for more precise and reliable segmentation of tree rings.

**Results and discussion:**

We show a performance increase in mean average recall of up to 18.7 percentage points compared to previously published results on the publicly available MiSCS (Microscopic Shrub Cross Sections) dataset. The newly added uncertainty estimation of our method allows for faster and more targeted validation of our results, saving a large amount of human labor. Furthermore, we show that panoptic quality performance on unseen species is more than doubled using multi-species training compared to single-species training. This will be another key step toward an AI-based version of the currently available ROXAS implementation.

## 1 Introduction

Tree rings are an outstanding archive for environmental research because of their absolute, annual dating precision and the wide occurrence of trees in many ecosystems around the globe (Fritts, 2001). The vast amount of applications in environmental research of tree-ring information can be largely grouped into the reconstruction of past variability and disturbances and the study of the impact of environmental variability and climate change on tree growth (Speer, 2010). Both perspectives are enabled by the interactions between trees and their environment, which modulate the amount and quality of wood formed in a given year.

There are different types of information stored in different parts of tree rings that are accessible through different methodological approaches (Frank et al., 2022). On the macroscopic scale, these methods range from tree-ring width measurement to measuring early- and latewood width, and to the relatively new method called blue intensity (Speer, 2010; Björklund et al., 2024). On the microscopic scale, they include tree-ring density based on measurements in x-ray images (Schweingruber et al., 1978) and high-resolution surface images (Rydval et al., 2024), and stable isotope composition in tree rings (Siegwolf et al., 2022).

Most recently, the quantitative wood anatomy (QWA) of tree rings (Fonti et al., 2010), also referred to as tree-ring anatomy or dendroanatomy, which measures cell dimensions from high-resolution digitized micro-sections or wood surfaces (von Arx et al., 2016), has been established. QWA excels at examining tree-ring properties at the cellular level due to its high resolution. Since the intra-ring position of cells corresponds to an intra-seasonal time window of cell formation (Fonti et al., 2010; Ziaco, 2020), investigations into sub-seasonal tree-environment interactions can be explored. The growing mechanistic understanding of the drivers, processes, and mechanisms of wood cell formation (e.g. Rossi et al., 2013; Cuny et al., 2014, 2019; Cabon et al., 2020; Peters et al., 2021; Silvestro et al., 2024) further contributes to linking components of cell structure to the corresponding cell formation processes (Carrer et al., 2017; Castagneri et al., 2017). Another very important asset of QWA is the structure-function link of xylem cells (Hacke et al., 2001, 2015). Structural properties of xylem cells define their function and inversely, tree responses to environmental variability impact the structural properties of xylem cells (Domec et al., 2008; Pittermann et al., 2011; Pauline S. et al., 2014; Wilkinson et al., 2015; Rosner, 2017; Guérin et al., 2020). Thus, several metrics related to water transport and carbon allocation can be derived from cell anatomical measurements (Fonti and Babushkina, 2016; Ziaco et al., 2016; Losso et al., 2018; Pacheco et al., 2018). The range of applications is wide and includes dendroclimatology and climate reconstructions (e.g. Ziaco et al., 2016; Björklund et al., 2020; Edwards et al., 2022; Seftigen et al., 2022; Björklund et al., 2023; Lopez-Saez et al., 2023); studies into wood biomass estimation (Cuny et al., 2015; Puchi et al., 2023), tree mortality (e.g. Hereş et al., 2014; Pellizzari et al., 2016; Klesse et al., 2020) and drought responses (Guérin et al., 2020; Olano et al., 2022; Buras et al., 2023); and forest ecology and climate sensitivity (Arnič et al., 2021; García-González and Souto-Herrero, 2023; Giberti et al., 2023) to name only a few. QWA relies on specialized software such as ROXAS (von Arx and Carrer, 2014; Prendin et al., 2017), WinCELL (Klisz, 2009), AutoCellRow (ACR) (Dyachuk et al., 2020) and CARROT (Resente et al., 2024), or adjusted general software such as QuPath (Keret et al., 2024) and ImageJ (see Scholz et al., 2013) to measure the numerous cells and rings visible in these thin sections.

Within the last decade, many advances in data acquisition and processing have been made, such as the usage of slide scanners instead of stitching single microscope images together (von Arx et al., 2016; Fonti et al., 2025). However, the fundamental software stack of ROXAS still relies on classical computer vision methods such as thresholding and edge detection for cell segmentation. These detected cells, and especially their sizes, are then used within strict given rules to predict the tree rings (von Arx and Dietz, 2005). This method poses several problems. First, insufficient cell segmentation results in poor tree-ring segmentation. In recent years, the performance of cell segmentation has drastically increased using deep learning methods (Garcia-Pedrero et al., 2020; Resente et al., 2021; Katzenmaier et al., 2023). These improvements will help the tree ring segmentation performance for some species, however, other species have a low number of cells or the cell sizes only differ slightly. For these species, better cell segmentation will still result in suboptimal segmentation performance.

More recently, deep learning-based approaches also tackled the problem of tree-ring segmentation by removing the dependency on cell segmentation and directly predicting tree rings based on the image itself. García-Hidalgo et al. (2024) showed promising results for European beech increment core images with linear ring structures by using a transformer-based UNet architecture to predict the tree-ring boundary. However, this method only predicts boundaries and not the whole ring area, making quantitative evaluation and comparison difficult.

Gillert et al. (2023) presented an openly accessible benchmark for circular ring segmentation in combination with a strong specialized baseline termed Iterative Next Boundary Detection (INBD), outperforming all evaluated general instance segmentation approaches. These circular tree rings are difficult to detect due to disappearing and reappearing rings, so-called wedging rings, and their concentricity. Additionally, standard instance segmentation approaches typically focus on compact objects and show poor performance on the large hollow rings included in the INBD dataset. Mask-R-CNN (He et al., 2017), a widespread instance segmentation approach, struggles to properly detect rings due to its two-stage approach of first detecting bounding boxes and, in the second segment, the content of the box. Since the bounding boxes of the rings overlap to a large degree, the non-maximum suppression fails. Contour-based methods such as Deep Snake (Peng et al., 2020) offer higher precision masks, however, they suffer from the same non-maximum suppression problem. Bottom-up approaches such as Multicut (Kappes et al., 2011) and GASP (Bailoni et al., 2022) detect smaller related pixel patches and cluster those patches together. These approaches perform better, however, they show deficits for disconnected rings and hard-to-detect boundaries. In comparison with these off-the-shelf computer vision algorithms, the INBD method proposed by Gillert et al. (2023) is tailor-made for concentric rings. It shows superior performance in ring boundary detection and better ring segmentation, even for discontinuous rings. The key features of the INBD approach are its iterative processing of the rings and its use of a polar grid instead of the cartesian grid typically used by off-the-shelf computer vision methods. We argue that the performance achieved by the INDB approach compared to standard methods illustrates how the very particular problem of tree-ring segmentation is best addressed with tailored methods.

In this paper, we build on these recent advances and propose a new circular ring detection model that achieves better performance with improved reliability. We follow the iterative paradigm of INDB but frame the boundary detection problem as a regression task. This leads to better segmentation performance and enables us to predict calibrated uncertainties on the boundary position. Additionally, we train our model in a multi-species setting and show higher performance on the known species and more robust predictions for unseen species.

## 2 Materials and methods

### 2.1 Dataset

**Figure 1:**
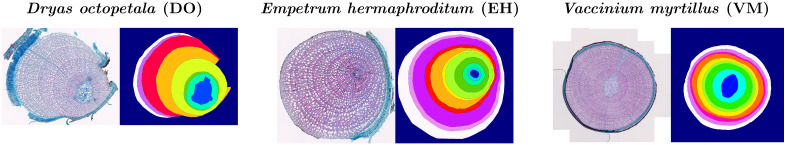
Example of the image and label pair for DO, EH, and VM from left to right. The image is publicly available in the MiSCS (Microscopic Shrub Cross Sections) dataset (Gillert et al., 2023).

In this study, we use the ring segmentation dataset MiSCS (Microscopic Shrub Cross Sections) introduced by Gillert et al. (2023). It contains *E* = 213 thin-section samples of arctic shrubs belonging to three different species: *Dryas octopetala* (DO), *Empetrum hermaphroditum* (EH), and *Vaccinium myrtillus* (VM). Each sample *i* ∈ [0*,E*] contains the input thin section image **X_i_** ∈ ℝ^3×^ *^H^* ^×^ *^W^* and the ground truth instance mask **Y_i_** ∈ [0*,e_i_*]*^H^* ^×^ *^W^*, with *e_i_* the number of rings in sample *i*. *Y_i_* assigns to each pixel position (*h,w*) of the image an integer value $y_{i}^{\left(h,\;w\right)}$ , identifying the specific ring to which the pixel belongs. A set of samples from the dataset can be found in **Figure 1**. The dataset’s images have a typical size of over 3,000 pixels per dimension. With a resolution of 2.27 pixel/µm, this results in sizes over 1,300 µm.

### 2.2 Method

#### 2.2.1 Overview

Our method predicts for each input image **X_i_** an instance mask ${\hat{\text{Y}}}_{i}\in {\left[0,n_{i}\right]}^{H\times W}$ , matching the ground truth segmentation *Y_i_* as accurately as possible.

As discussed in the introduction, conventional computer vision approaches for instance segmentation tend to struggle with the specific challenges of ring segmentation. We therefore build on recent work by Gillert et al. (2023) and adopt a tailored approach that addresses the task iteratively, i.e., ring by ring. Our model processes the input image *radially* from pith to bark and regresses the distance to the next ring from the previous ring.

We present an overview of our method, named INBD-R, as pseudo-code in **Algorithm 1**. The main components are as follows. A trainable **semantic segmentation model** processes the downscaled input image and is followed by the iterative model. The semantic segmentation model returns the location of the *pith* from which the iterative process starts, as well as a first *estimation of the ring width*. Next, starting from the position of the pith, the position of the next ring is iteratively predicted. Each iteration includes the following steps:

- To leverage the circular geometry of thin-slice images, the input image is projected onto a polar grid with the origin in the center of the pith prediction.
- The polar image is radially cropped based on the estimated ring width.
- The cropped polar image is processed by a **trainable radial regression** model that predicts the precise position of the next ring and an uncertainty value for the ring position.

Algorithm 1 **Pseudo-code for our iterative boundary segmentation method. Blue represents trainable models and red non-trainable processing.**

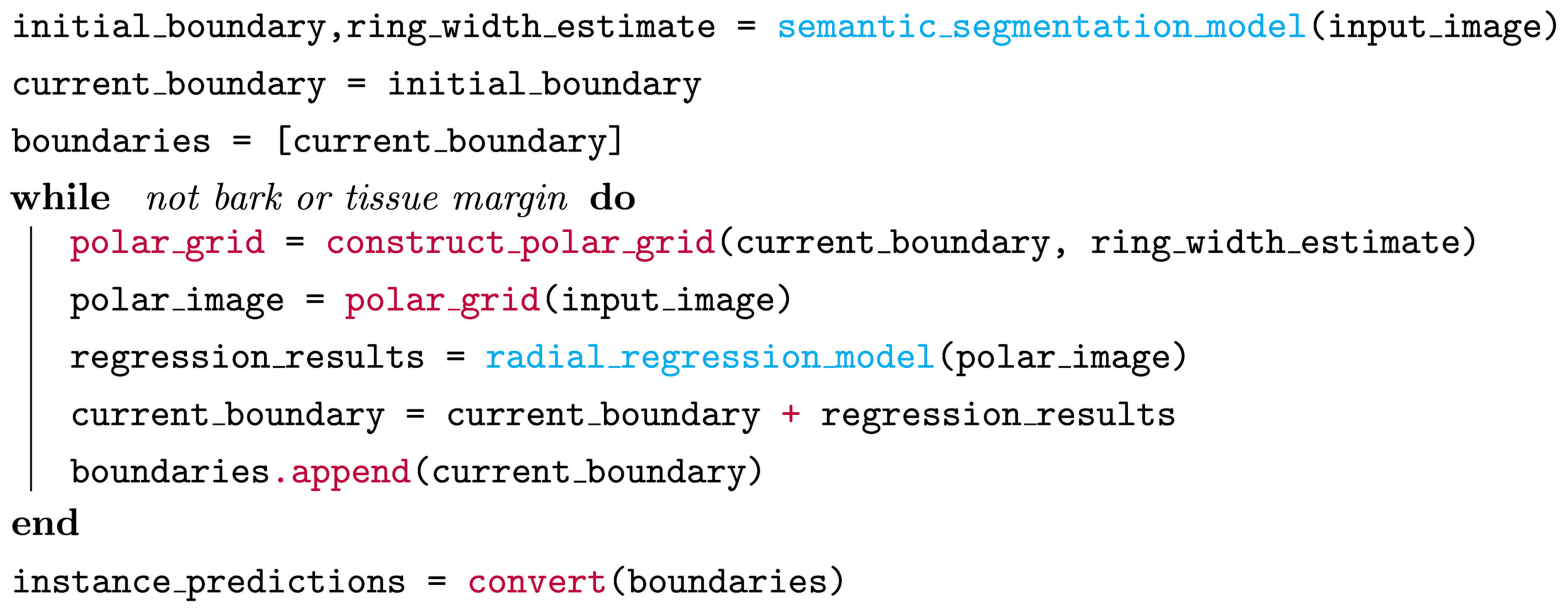

**Figure 2:**
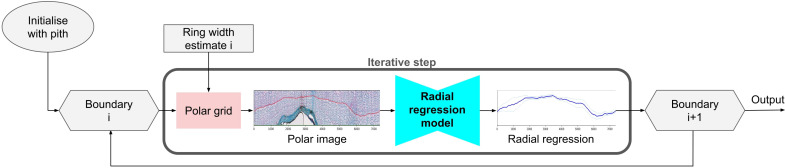
Overview of the iterative process. We initialize the boundary with the pith prediction from the semantic segmentation network. Based on this boundary and the ring width estimate from the semantic segmentation model, we create the polar grid to interpolate the polar image. We feed this image to the radial regression model, yielding the radial regression prediction, which we use to predict the next boundary. With this boundary, we start the next iteration. This process repeats until the end of the xylem is reached.

The iterative process, visualized in **Figure 2**, ends when the bark or the edge/outline of the xylem tissue is reached, which is detected via the missing ring width estimate. After the iterative process, the ring positions are converted back to instance masks by drawing their polygons from outside to inside. To gain prediction at full resolution, the continuous polygon points are upscaled. We first train the semantic segmentation model on the data. In the second step, we use the trained model to preprocess the input data to prepare it for the training of the radial regression model. Third, we train the radial regression model using our fast iterative unrolling training procedure. We describe each part of INBD-R in further detail in the following sections.

#### 2.2.2 Semantic segmentation model

**Figure 3:**
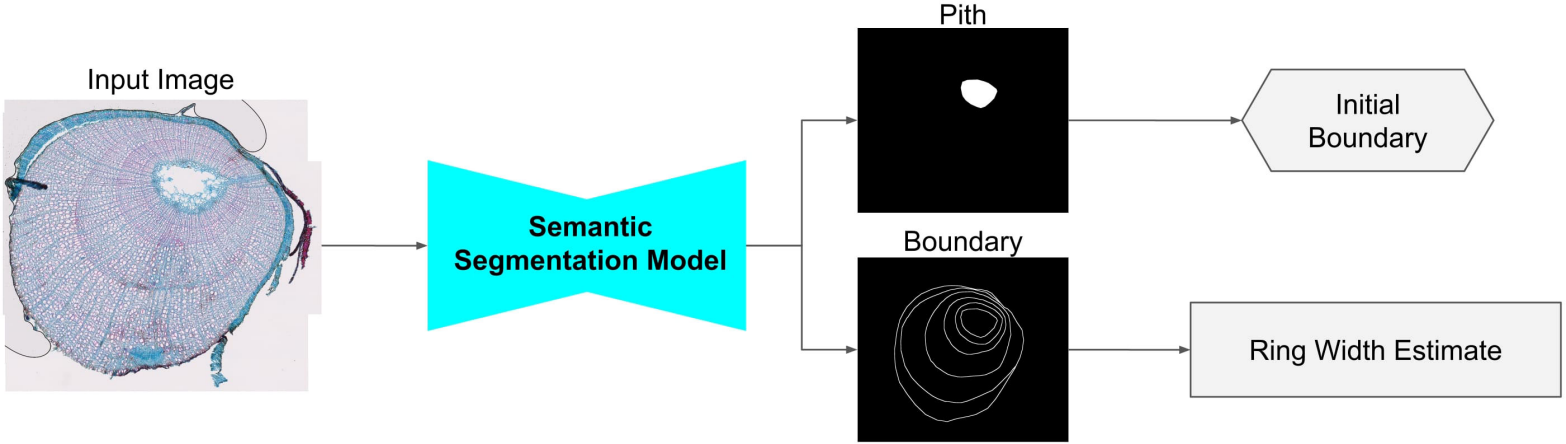
Overview of the semantic segmentation model. The input is passed through the semantic segmentation model, predicting binary masks, which are converted to the initial boundary and a ring width estimate.

**Task**: The semantic segmentation model aims to find the position of the pith and produce a first estimate of each ring’s width, as visualized in **Figure 3**. Following Gillert et al. (2023), we achieve this through a semantic segmentation step, where each pixel is classified within the three classes:

- Pith: center of the thin section
- Boundary: pixels at the interface between two consecutive rings
- Background: pixels not containing xylem or pith tissue

The boundary class itself contains most of the target information. However, the boundary prediction as a final result is not a viable option since a one-pixel-wide boundary prediction cannot be easily converted to instance masks. This is because of the insufficient robustness to wrong predictions, struggles with hard-to-detect boundaries on small scales, and the possibility of connecting wedging rings.

We use a convolutional neural network with sigmoid activation in the final layer to predict one binary mask for each of the four classes. To reduce the computational load and to increase the spatial context, the semantic segmentation model operates on a ×4 downscaled version of the input image.

**Architecture and training**: We employ a UNet (Ronneberger et al., 2015) with a Res2Net (Gao et al., 2021) backbone and train it with a binary dice loss (Sudre et al., 2017) defined in Equations 1, 2. This loss is applied to each mask individually. Therefore, *y* represents the ground truth binary mask and $\hat{y}$ the predicted binary mask.


(1) $$L_{\text{Dice}}\left(y,\hat{y}\right)=1-\frac{\sum \left(y\cdot \hat{y}\right)+\epsilon }{\sum \left(y+\hat{y}\right)+\epsilon }$$

(2) $$L=\alpha_{0}L_{\text{Pith}}+\alpha_{1}L_{\text{Background}}+\alpha_{2}L_{\mathrm{Boundary}}$$

**Figure 4:**
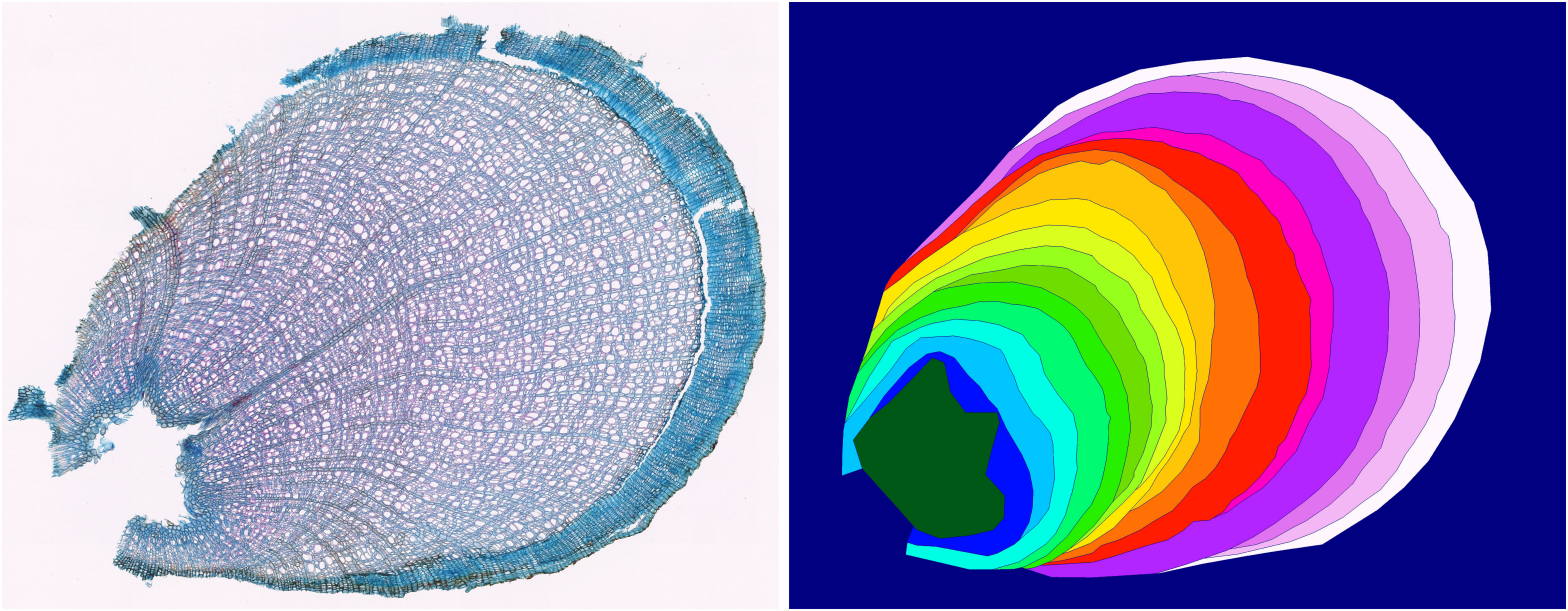
Image and label pair from the dataset by Gillert et al. (2023) where the pith has been torn off by the thin-sectioning process. Additionally, the xylem is interrupted on one side so that the pith and background labels touch.

The binary dice losses are weighted with *α*_0_ = 0.1, *α*_1_ = 0.01, and *α*_2_ = 1. Binary segmentation per class is superior to multi-class cross-entropy training because it prevents the model from exclusively deciding between the background and the pith since they can look similar if the pre-processing of the xylem damaged the pith tissue, as seen in **Figure 4**.

#### 2.2.3 Polar grid

**Initial boundary position**: We convert the predicted binary mask of the pith into the initial boundary position. This position is defined by the center point of the binary mask and equally spaced outer edge points of the mask of shape ℝ^2×^ *^M^*. *M* represents the adaptive angular resolution, which grows proportionally to the distance to the center, ensuring similar spacing between boundary points across ring positions. The center point is reused for all boundaries. The actual value *M* for the next ring is set to 2*π* times the average distance between the center and the current ring boundary.

**Polar transformation**: The downsampled input image is converted into an image in polar space (polar image) where the upper row of pixels corresponds to the current boundary. The current boundary equals the initial boundary in the first iteration and is updated with the next predicted boundary in each iteration step. The rough estimate for the next ring width *P* ∈ ℝ is computed based on the current ring and the binary mask for the *boundary* class of the semantic segmentation model. *P* is obtained by taking 1.5 × the 95th percentile of the distances from the current ring to the next boundary pixel in a radial direction evaluated at each boundary point within the current ring. This overcomes outliers and ensures that the next ring boundary is within the polar image. Further details on the angular resolution *M* and the rough ring width estimate *P* can be found in the work by Gillert et al. (2023). The polar image is constructed by interpolating the downsampled image on a polar grid of shape *N* × *M*, with *N* = 256 the number of points in a radial direction. These points start at the current boundary and fan out in a radial direction with a distance of *P/N* between the points. We generate the polar image of shape ℝ^6×^ *^N^* ^×^ *^M^* by interpolating the down-sampled image as well as the background and boundary predictions without applying the activation function of the semantic segmentation model. For the 6th channel, we calculate the distance from each interpolation point to the center and normalize its values from 0 to 1. This helps the model to understand jumps in the previous boundary and gives information on the global scale.

#### 2.2.4 Radial regression model

**Figure 5:**
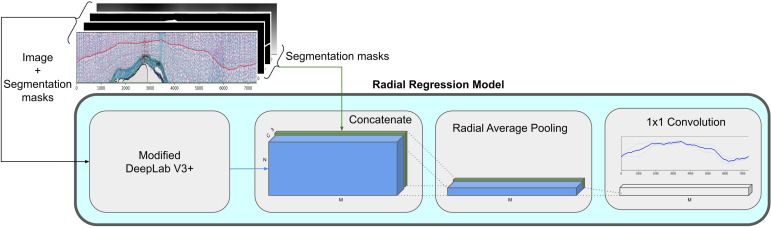
We feed the polar image through a DeepLabV3+ (Chen et al., 2018) model and it is concatenated with the feature dimensions of the polar image. We follow this with a radial average pooling which reduces the height dimension to one. A final 1x1 convolution is used to get to our final radial regression.

**Regression**: Different from previous work (Gillert et al., 2023), we frame the prediction of the next boundary prediction as a regression task. To this end, we employ a second deep net, the radial regression model, visualized in **Figure 5**. It predicts a real-valued distance to the next boundary for each of the *M* angular positions of the polar grid.

Addressing this problem as a regression task has several decisive advantages. First, it completely alleviates ambiguous predictions occurring with a segmentation approach such as predicting multiple edges within a pixel column, described by Gillert et al. (2023). In other words, our approach enforces *by-design* that only one boundary is predicted at each step. A second advantage is that the architecture implicitly enforces a smoothness constraint on the ring width because it uses bilinear upsampling in the segmentation head. Third, our design also makes the model predictions directly interpretable as they correspond to angular ring widths. In particular, this enables us to additionally predict an uncertainty value for the ring position, as described in Section 2.2.5.

**Architecture and training**: We use the DeeplabV3+ (Chen et al., 2018) architecture with a fast MobileNet (Howard et al., 2017) backbone as a basis and modified it. First, we reduce the number of convolution filters from 256 to 64 in the altrous spatial pyramid pooling module of the DeeplabV3+. Second, we remove the normalization layer after the atrous spatial pyramid pooling. Third, we convert the convolutions into circular convolutions proposed by Peng et al. (2020). These convolutions wrap around in angular direction, which accounts for the circular polar space. Finally, we replace the batch norm layers (Ioffe, 2015) with instance norms (Ulyanov et al., 2016).

The change to instance norms is necessary since the circular convolutions in combination with the adaptive angular resolution *M* prevent conventional batching of input samples by concatenating them along the batch dimension due to shape mismatches. The lack of batching prevents the use of batch norm layers. To emulate batch training, we use gradient accumulation, collecting gradients of multiple forward passes before the weights update. The normalization layer after the atrous spatial pyramid pooling had to be removed because of the necessary change from batch to instance norm.

We add a bypass for the features from the semantic segmentation model by concatenating them to the output of the previously described modified DeeplabV3+, as visualized in **Figure 5**. This allows the further use of the already processed features from the semantic segmentation model. Since concatenations does not change the spatial shape, we still have the same shape as the input *N* × *M* polar image. We reduce the height dimension to one with a radial average pooling with a kernel of shape *N* × 1. Finally, we apply a 1 × 1 convolution to reduce the channel dimension to one, and output the predicted distances to the next ring at each angular position **Δ** = [*δ*_1_,···*,δ_M_*] ∈ ℝ*^M^*.

We train the radial regression model with L1 loss. We find that the optimization is more stable if the target distances is first normalized to values in [−1,1] as follows:


(3) $$n\left(d\right)=\frac{d-\frac{N}{2}}{\frac{N}{2}}$$

Hence our radial regression model is trained by minimizing (Equation 4)


(4) $$L_{1}=\sum_{m=1}^{M}\left|\delta_{m}-n\left(d_{m}\right)\right|$$

with **D** = [*d*_1_,···*,d_M_*] ∈ ℝ*^M^* the ground truth distances to the next ring. To encounter every ring with a similar frequency in training, we initialize the iterative process with every ground truth ring boundary as a starting point and limit the number of iterations to *K* = 3.

#### 2.2.5 Uncertainty estimation

In the previous work of Gillert et al. (2023), the prediction of the next boundary is cast as a pixelwise segmentation problem of the polar image. In our work, instead of pixelwise class scores, we regress a distance for each angular position *m*. Hence, the predictions returned by our model are straightforward to understand. This also enables us to train our model to predict an uncertainty value that is directly expressed in terms of ring width, instead of a more abstract uncertainty on each pixel’s class prediction. For this, we modify the final one-by-one convolution of our radial regression model to predict two parameters instead of one: in addition to the distance **Δ,** the model also outputs uncertainty values for each radial position: **B** = [*b*_1_,···*,b_M_*] ∈ ℝ*^M^*. We train these predictions using a Negative Log Likelihood (NLL) with Laplacian distribution, as recommended by Yeo et al. (2021), meaning that *δ* and *b* are interpreted as the mean and scaling parameter of a Laplace distribution (Equation 5):


(5) $$f\left(x|\delta ,\;b\right)=\;\frac{1}{2b}\text{exp}\left(-\frac{\left|x-\delta \right|}{b}\right)$$

and both are supervised using the following loss function (Equation 6):


(6) $$L_{NLL}=\sum_{m=1}^{M}\frac{\left|\delta_{m}-n\left(d_{m}\right)\right|}{b_{m}}+log\left(b^{m}\right)$$

This loss enables the model to predict a higher uncertainty *b* for samples where the regression error is hard to minimize, and thus reduce their impact on the overall loss at the cost of increasing the second term. After convergence, the model learns to predict higher uncertainty *b* only for samples for which the error is more likely to be high. The predictive uncertainty *b* is the scaling factor of the Laplace distribution expressed in normalized units, we transform it to a standard deviation *σ_m_* of the distance expressed in original units as follows (Equation 7):


(7) $$\sigma_{m}=\sqrt{2}\cdot b_{m}\cdot \frac{P}{2}$$

### 2.3 Training and implementation details

#### 2.3.1 Training

**Dataset splits**: We follow the given training and testing splits, resulting in only 22, 24, and 22 training samples with average diameters of 3,700, 3,260, and 3,979 pixels for DO, EH, and VM. Example images and ground truth labels can be seen in **Figure 1**.

**Semantic Segmentation Model**: We train this model for 1,000 epochs using a cosine annealing learning rate schedule with a starting learning rate of 1*e* − 3 and an end learning rate of 1*e* − 5. The samples are augmented with random scaling, rotation, flipping, and color jitter and cropped to a size of 512. We apply the standard ImageNet pixel normalization. These samples are stacked into batches of size 8. For the backbone, we use the default hyperparameters and pre-trained on ImageNet.

**Radial Regression Model**: We train our radial regression model for 500 epochs with a cosine annealing learning rate schedule starting at 1*e* − 3 and ending at 1*e* − 5. As augmentation, we use color jitter since the other augmentations we used for the semantic segmentation model do not work in polar space. We apply the standard ImageNet pixel normalisation. As described in section 2.2.4, we emulate the batch size of 8 with gradient accumulation.

#### 2.3.2 Iterative unrolling

In the original implementation by Gillert et al. (2023), a training step consists of running the semantic segmentation model, *K* = 3 consecutive iterative steps, which include polar grid construction and interpolation and running the regression model.

We propose a more efficient implementation that enables faster training. Our efficient implementation is based on 1) saving the predictions of the segmentation model to disk instead of re-running it at each epoch and 2) unrolling the iterative steps onto different epochs. Indeed, we identified the polar grid construction and interpolation as the main bottleneck in the training process.

The polar grid construction cannot be done in an offline pre-processing step since it depends on the predicted ring of the previous iterative step. However, we can offload the polar grid construction and interpolation to other threads, allowing for parallelizability during radial regression model training. To achieve this, we propose a new training process named *iterative unrolling*. We unroll the iterative steps over *K* epochs. In iterative unrolling, we only run one iterative step per training step. However, we require the main thread to save the predicted ring to disk in the current epoch. In the next epoch, this boundary will be read by the data loader, which calculates the polar grid and interpolates the next polar image. Therefore, this sample is effectively in the next iterative step for this epoch. Splitting the iterative process over multiple epochs avoids race conditions between saving and loading the boundary files. After 3 epochs, we start over with the ground truth ring, as is done in the original implementation.

**Figure 6:**
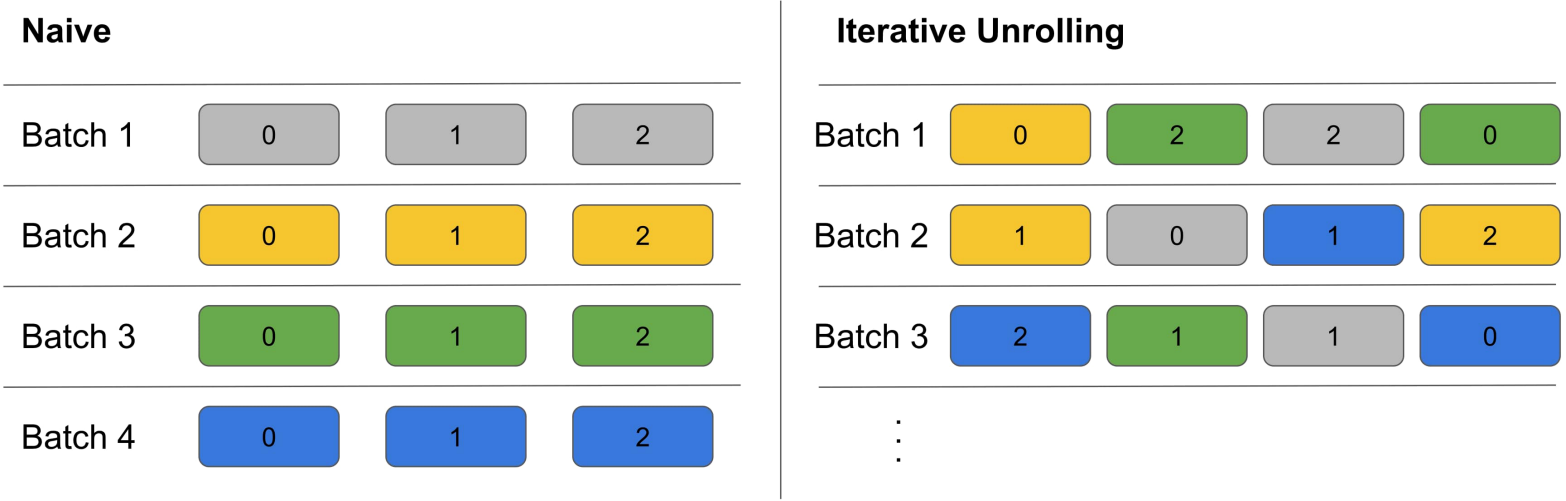
Comparison of batch construction for the naive implementation and the iterative unrolling. Each block represents a polar image. Colors indicate the starting ring and numbers indicate the iteration step. For example, all yellow belongs to one input image and the number indicates which iteration the individual polar image belongs to. The naive implementation has only the same input image in a sample. Iterative unrolling randomly mixes input samples and iteration steps. Additionally, iterative unrolling allows for setting the batch size independent from the iteration depth.

In the original iterative implementation, the model sees each sample *K* times per epoch. To mimic this, we duplicate each sample *K* times and start the iterative process in a staggered manner where the *i*-th copy starts in the *i*-th epoch using the predicted values. This allows for the duplicate samples to be in different steps in the iterative process. Another advantage of iterative unrolling is the possibility to gain batches with polar images from many different input images, as visualized in **Figure 6**. This is more difficult in the original implementation due to the *K* steps with the same input image. These more diverse batches result in more stable gradients, which is especially useful for multi-species training where images of different species display larger diversity. Besides the more stable gradients, we achieve a speedup of two to three times using four parallel threads.

### 2.4 Metrics

To estimate the performance of an instance segmentation approach a measurement of mask similarity is necessary. The most common mask similarity measurement is the Intersection over Union (IoU) also referred to as the Jaccard index. It is defined as Equation 8.


(8) $$IoU\;\left(y,\hat{y}\right)=\;\frac{\left|y\cap^{\;}\hat{y}\right|}{\left|y\cup^{\;}\hat{y}\right|}$$

Naively calculating the average IoU between each prediction mask and all label masks will not result in a desired metric. Gillert et al. (2023) proposed to use the mean Average Recall (mAR) [Equation 9] by Hosang et al. (2015) and Adapted Rand errors (ARAND) [Equation 10] by Arganda-Carreras et al. (2015) for this dataset. These two metrics effectively measure the performance but are less intuitive. Therefore, we additionally evaluate the methods with the Panoptic Quality (PQ) respective to its two parts Segmentation Quality (SQ) and Recognition Quality (RQ) introduced by Kirillov et al. (2019).


(9) $$mAR=2\int_{0.5}^{1}\mathrm{recall}\left(o\right)\;do=\;\frac{2}{k}\sum_{i=1}^{k}max\left(IoU\left(gt_{i}\right)-0.5,\;0\right)$$

(10) $$ARAND=1-\frac{\sum_{ij}p_{ij}^{2}}{\alpha \sum_{k}s_{k}^{2}+\left(1-\alpha \right)\sum_{k}t_{k}^{2}}$$

PQ, SQ, and RQ are defined in Equation 11 and give a good overview of the performance. SQ gives an intuition on how well instances with an *IoU >* 0.5 are segmented and RQ measures how many instances are matched.


(11) $$PQ=\underset{\text{Segmentation Quality}\left(\text{SQ}\right)}{\underset{︸}{\frac{\sum_{\left(y,\hat{y}\right)\in TP}IoU\left(y,\hat{y}\right)}{\left|TP\right|}}}\times \frac{\left|TP\right|}{\underset{\text{Recognition Quality }\left(\text{RQ}\right)}{\underset{︸}{\left|TP\right|+\frac{1}{2}\left|FP\right|+\frac{1}{2}\left|FN\right|}}}$$

These metrics are instance-based and do not give any intuition of how far the ring boundary is from the ground truth label. To further increase interpretability, we introduce a new metric that measures the ring segmentation error in pixels because the scaling between pixel and *µm* is not provided with the dataset.

To measure the error, we first match prediction and ground truth instances with *IoU >* 0.5, similar to PQ. Once the matches are established, we calculate the minimum distance from each boundary point of the prediction to the closest boundary pixel of the label. This is formally stated in Equation 12 where *AE_i_* represents the absolute error for predicted boundary points ${\hat{y}}_{i}$ and the label boundary pixel *y_j_*. We use these absolute errors to calculate the mean Absolute Error (MAE) and the medium Absolute Error (MedAE).


(12) $$AE_{i}=\underset{j}{\min}\|{\hat{y}}_{i}-y_{i}\|$$

For the evaluation of the uncertainty estimation, we use the Expected Normalized Calibration Error (ENCE) introduced by Levi et al. (2022). It is defined in Equations 13–15 and estimates the calibration of the uncertainty, where *σ* is the unnormalized standard deviation and *µ* is the predicted mean. The RMSE is calculated in the same manner as the MAE and MedAE using the instance matching beforehand.


(13) $$mVAR\left(\alpha \right)=\;\sqrt{\frac{1}{\left|B_{a}\right|}\sum_{t\in B_{a}}\sigma_{t}^{2}}$$

(14) $$RMSE\left(a\right)=\;\sqrt{\frac{1}{\left|B_{a}\right|}\;\sum_{t\in B_{a}}{\left(\underset{j}{\min}\|\mu_{t}-y_{j}\|\right)}^{2}}$$

(15) $$ENCE=\;\frac{1}{O}\sum_{n=1}^{O}\frac{\left|mVAR\left(a\right)-RMSE\left(a\right)\right|}{mVAR\left(a\right)}$$

The ENCE formula uses binning according to the predicted uncertainty. Therefore, the samples are separated into *U* bins where *B*_1_ contains the *Q* samples with the lowest uncertainty and *B_U_* with the highest uncertainty. *Q* = *T/U* where *T* is the number of predicted boundary points and we set *U* to 100 for our experiments.

## 3 Results and discussion

### 3.1 Competing approaches

The main competing approach we compare to is INBD (Gillert et al., 2023), as it was superior to all other approaches in their experiments on the same dataset. We report the performance of the INDB model as implemented in the original paper of Gillert et al. (2023). For a fair comparison, we also report the performance of an INBD variant with the same segmentation backbone as ours and with tuned hyperparameters.

Next, we report the performance of four variants of our approach:

- **INBD-R**: with L1 loss and trained on a single species
- **INBD-Ru**: with uncertainty estimation and trained on a single species
- **INBD-Rm**: with L1 loss and multi-species training
- **INBD-Rum**: with uncertainty estimation and multi-species training

Note that we report the average metric over three different runs to ensure the stability of our results, given the small dataset size. This explains why the numbers reported for INBD do not exactly match those of Gillert et al. (2023), but they are consistent with their reported error bars.

### 3.2 Main results

We report the performance of the different models on the three species of the dataset in **Table 1**. Overall, our proposed INBD-R outperforms the previous best existing approach by a large margin, ranging from 7.4% to 18.7% for mAR and 8.4% to 23.5% for PQ, depending on the species. Our results show that our approach significantly improves the state of the art for ring detection in anatomical images.

**Table 1:** Results of the method comparison.

	Method	mAR↑	ARAND↓	PQ↑	SQ↑	RQ↑	MAE↓	MedAE↓	ENCE↓
EH	INBD	0.760	0.100	0.783	0.861	0.893	8.71	2.81	–
	INBD tuned	0.788	0.091	0.802	0.884	0.908	8.87	2.56	–
	INBD-R	0.823	0.077	0.837	0.897	0.932	6.91	2.49	–
	INBD-Ru	0.823	0.075	0.844	0.897	0.940	6.51	2.42	0.973
	INBD-Rm	**0.842**	**0.072**	**0.867**	**0.906**	**0.951**	5.90	2.35	–
	INBD-Rum	0.832	0.074	0.855	0.902	0.948	**5.76**	**2.31**	0.699
DO	INBD	0.573	0.183	0.616	0.800	0.770	20.1	8.24	–
	INBD tuned	0.727	0.120	0.709	0.854	0.830	14.9	5.57	–
	INBD-R	**0.760**	**0.103**	**0.797**	0.862	**0.925**	13.2	5.85	–
	INBD-Ru	0.755	0.107	0.792	0.861	0.920	**13.0**	5.79	4.57
	INBD-Rm	0.746	0.110	0.769	**0.867**	0.887	14.8	5.52	–
	INBD-Rum	0.751	0.108	0.785	0.865	0.907	16.3	**5.44**	7.17
VM	INBD	0.688	0.121	0.608	0.872	0.697	16.6	3.64	–
	INBD tuned	0.791	0.076	0.724	0.902	0.803	10.2	2.60	–
	INBD-R	0.826	0.061	0.795	0.907	0.877	7.96	2.66	–
	INBD-Ru	0.821	0.062	0.790	0.910	0.868	7.42	2.45	0.582
	INBD-Rm	**0.853**	**0.054**	0.839	**0.916**	0.917	7.37	2.38	–
	INBD-Rum	0.848	0.055	**0.843**	0.915	**0.921**	**6.36**	**2.22**	0.753

The addition of u and m to our INBD-R method stands for the addition of uncertainty and multispecies training, respectively. INBD is the method proposed by Gillert et al. (2023). These numbers slightly differ from those previously published since we reran their method to generate an average of three runs. However, the average falls into the standard deviation provided in their paper. INBD tuned is further tuned with our semantic segmentation model and additional hyperparameter tuning. Arrows indicate values of higher performance. Bold highlights the best performance and underlined highlights the second best performance.

**Figure 7:**
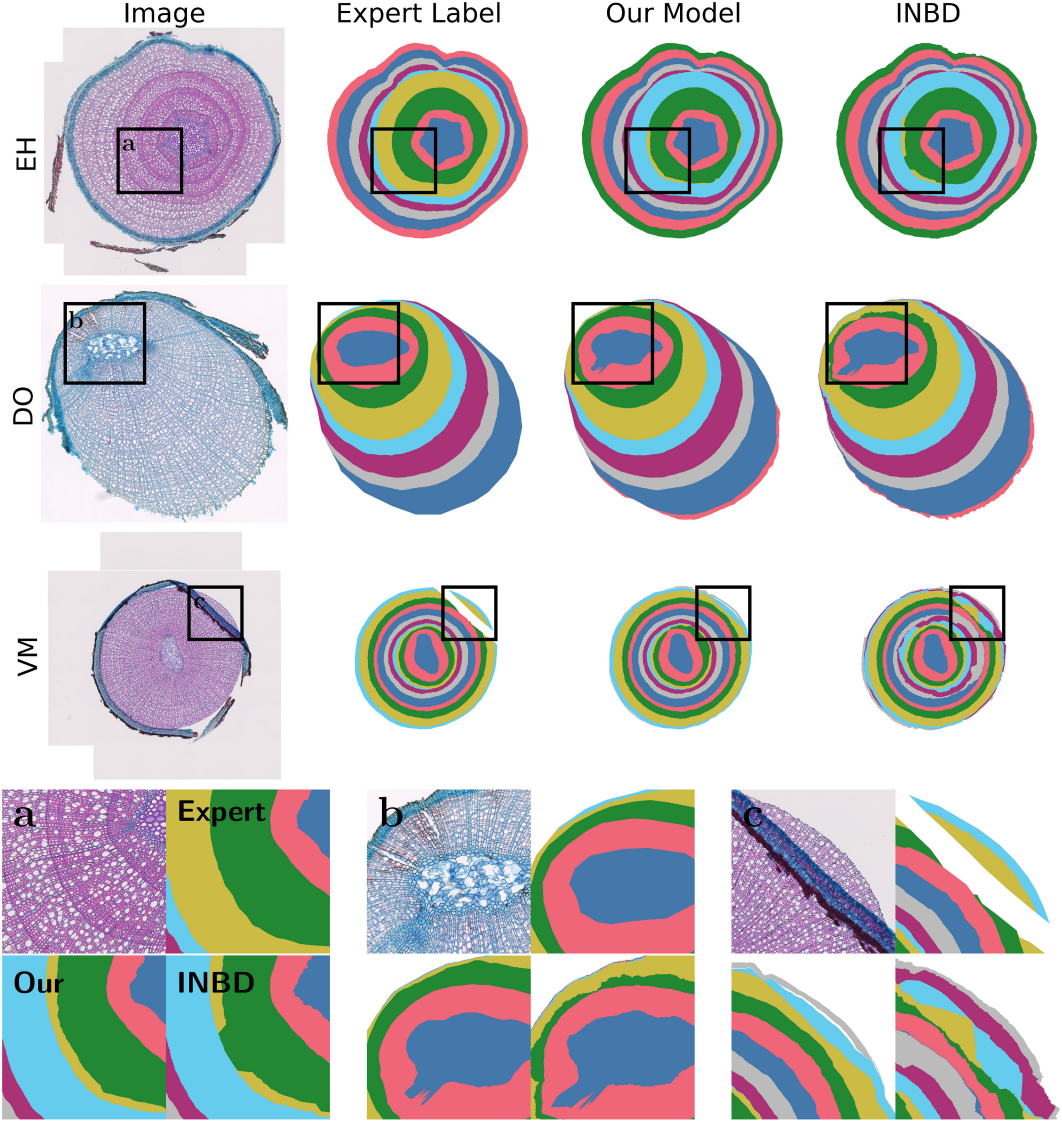
Visual results for ring segmentation. Each row shows, from left to right, the original anatomical image, expert label, and the model predictions using our approach and the INBD approach. **(a)** highlights the additional ring added by both our model and the INBD model. The comparably larger vessel lumina in this region resembles the characteristics of an additional ring. This demonstrates the difficulty of accurately determining the ring boundaries in a single image. **(b)** highlights a region from where an adventitious root is originating, which the deep learning models mistake as extended pith. Additionally, it can be seen that distinguishing between rings is more difficult for narrow rings, but our model’s prediction remains closer to the expert annotation. **(c)** shows different results for the region where the bark is folded over the xylem. We can clearly see the jump between rings of INBD [also visible in **(a)**]. Our results show a ring completed with a similar width (green), which increases the robustness of ring width estimation compared to the expert label, while the INBD predicted unrealistic rings.

**Comparison with INBD**: More specifically, **Table 1** shows improvements of 3.5 pts for mAR, 1.7 pts for ARAND, and 7.4 pts for PQ for our single species model averaged over all species compared to the tuned INBD model. This performance increase from tuned INBD to INBD-R is solely from the reformulation from segmentation to regression. The improvement is not only visible in the metrics but also visually apparent, as seen in **Figure 7**. There are several cases where INBD jumps between two different rings, resulting in unnatural tree ring results. Our regression approach, in contrast, displays smooth rings even in cases where this is not directly visible. **Figure 7** additionally shows the difficulty in segmenting rings on a single image since both algorithms show in the top two rings plausible additional rings for which experts need additional input to find a definite answer. Besides the instance-based metrics, our approach also improves on the more interpretable MAE and MedAE metrics, which directly show the mean and median distance between the predicted and ground truth ring boundary. The differences in these metrics seem small, however, since these metrics are only calculated for detected rings. Our approach shows lower offsets even though it includes the more difficult rings. This difference in included rings is displayed by the RQ metric for which our approach shows a significantly higher performance of up to 12 pts. This is especially impressive if we take the resolution of 2.27 pixel/µm into account, as then the median absolute error becomes only 2.5 µm for DO and 1 µm for EH and VM.

**Multi-species training**: Training our method on all species instead of on a single one shows further performance improvement of 2.7 pts for VM and 1.9 pts for EH with only a slight decrease of 1.4 pts for DO, but still outperforming the tuned INBD by a significant margin. This performance increase can be attributed in part to the increased amount of training data, however, it also forces the model to focus on more general concepts that apply to more than one species. These more general concepts can then be easily transferred to unseen species, as we demonstrate in the next section. In section 3.3.3, we further investigate the performance differences between the species.

**Figure 8:**
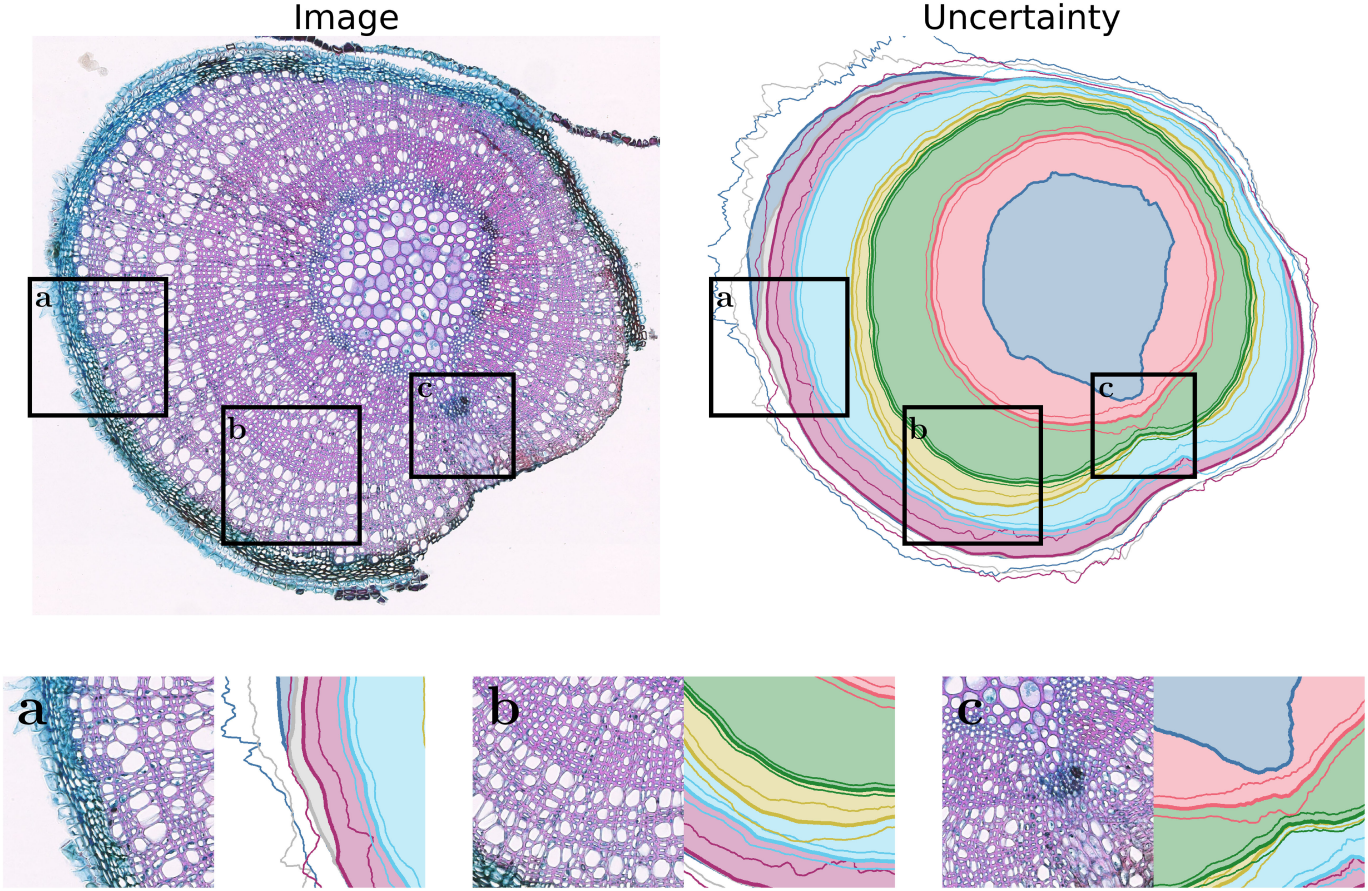
Visualization of the uncertainty. The additional lines display one standard deviation estimated by the model. **(a)** displays problematic large uncertainties for ring boundaries close to the bark. One reason for this is the low amount of training data for such cases. **(b)** shows, as desired, increased uncertainty where the ring boundary is difficult to detect. **(c)** shows a case where the pith bulges outwards, making exact ring detection more difficult and, therefore, resulting in increased uncertainty.

**Uncertainty estimation**: Adding uncertainty estimation to our model does not affect the segmentation performance significantly. The performance decrease is less than 0.5 pts for mAR and 0.5 pts for ARAND. For PQ, we can see a performance change from −0.5 pts to +0.7 pts. The uncertainty prediction can be used to focus manual validation and editing to uncertain areas to ultimately decrease the amount of human labor needed to validate and further improve the measurements of our method. **Figure 8** visualizes the predicted instances with their uncertainty. It turns out that our method has a small uncertainty for clearly visible rings and larger uncertainties for areas with many smaller rings where jumps between ring boundaries are more probable.

#### 3.2.1 Generalization to unseen species

One of the benefits of our efficient implementation of the iterative detection is that it enables multi-species training. In this section, we showcase how multi-species training leads to better generalization to species *not seen* in the training data.

To measure the better generalization, we compare our multi-species model to a species-specific model on images from unseen shrub and tree species. These samples belong to *Salix polaris*, *Fagus sylvatica*, *Fraxinus excelsior*, and *Vaccinium vitis-idea*. All samples are of markedly different quality to those in the training set as they were produced in different labs using different equipment and slightly different protocols. Due to the large visual differences, no method was able to detect the pith. Therefore, we provide the methods with the ground truth pith, which is an acceptable amount of user input if the subsequent automatic ring segmentation is of sufficient quality.

The results in **Table 2** show clear improvement for the multi-species method, surpassing the single-species methods by more than 10 pts for mAR, 10 pts for ARAND, and 20 pts for PQ. For MAE and MedAE, we observe high values in all cases, but the multi-species values are clearly smaller. This is especially impressive since the higher RQ value of 0.544 indicates that more rings are included for the MAE and MedAE calculations. These additional rings include rings that were too difficult to detect for the single species model. The displayed performance improvements are achieved by a larger and more diverse dataset. Nonetheless, the multi-species training still contains only 68 images, which explains the large performance drop for unseen species. Further diversifying and increasing the training set will reduce the number of completely unseen species and allow for better generalization of our model.

**Table 2:** Ring detection performance for unseen species [*Fagus sylvatica* (14 images from two different datasets), *Fraxinus excelsior* (3), *Salix polaris* (10)].

Method	mAR↑	ARAND↓	PQ↑	SQ↑	RQ↑	MAE↓	MedAE↓
Single (DO)	0.318	0.503	0.207	0.810	0.256	95.6	13.3
Single (EH)	0.323	0.558	0.149	0.810	0.184	48.1	9.59
Single (VM)	0.376	0.423	0.212	0.833	0.254	92.8	17.9
Multi (DO, EH, and VM)	**0.504**	**0.285**	**0.434**	**0.798**	**0.544**	**48.5**	**8.49**

The names in brackets show which species the model was trained on. Bold highlights the best performance. Arrows indicate values of higher performance.

**Figure 9:**
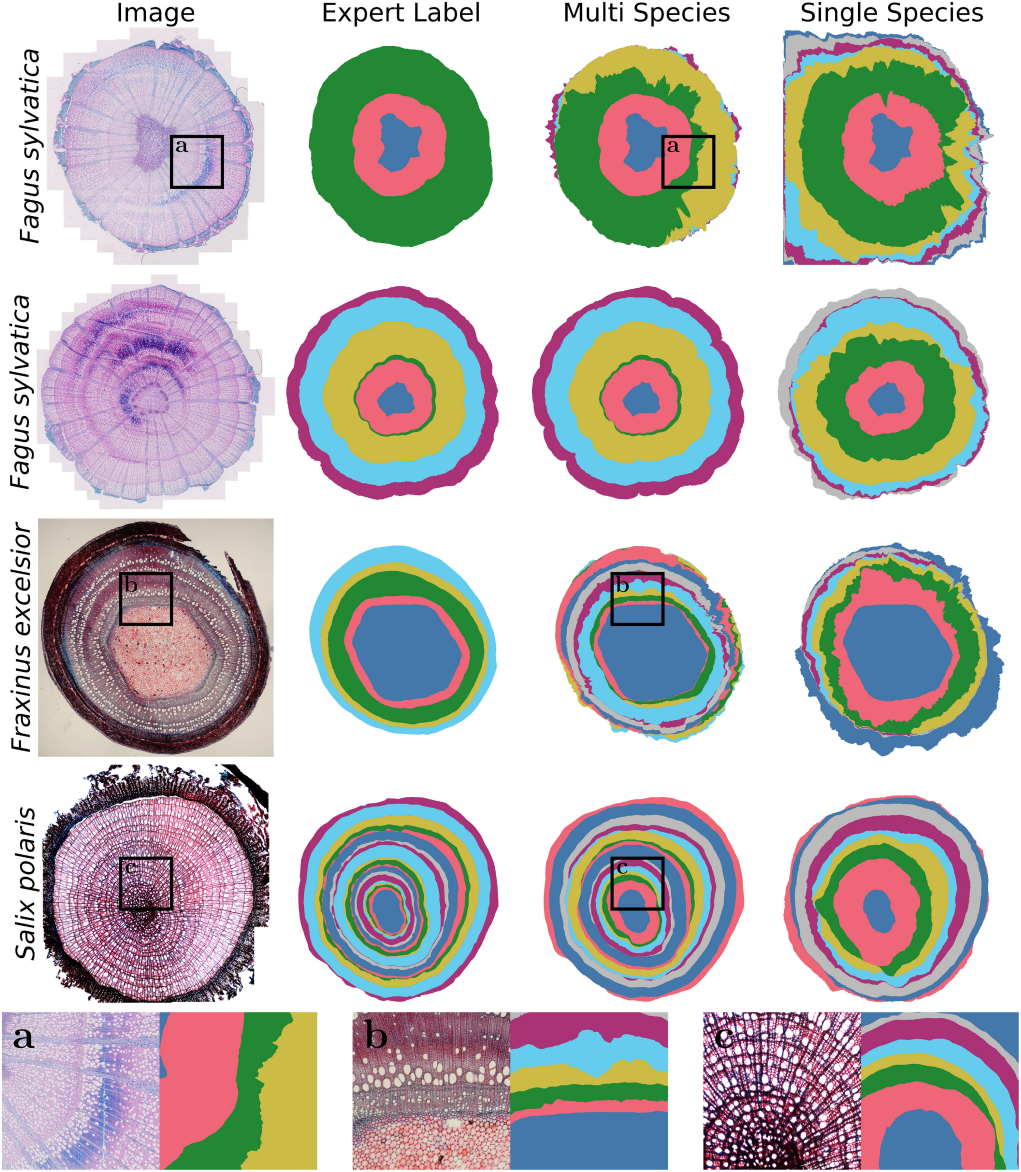
Visual results on unseen species. Each row shows, from left to right, the original anatomical image, expert label, and the model predictions using multi- and single-species (in this figure: VM) training. The improved performance between multi- and single-species training is clearly visible. However, different qualities of ring detection are visible for similar images (first and second row). **(a)** shows visually different-looking rings within a sample that the model predicts wrongly. **(b)** shows additional false rings that were detected because of lines of comparably wide vessels in this ring-porous species that did not match any of the diffuse-porous training species. **(c)** shows cases where narrow rings are not always properly detected, although dark tangential bands indicate the rings well. All these properties were not present in the training species, explaining the difficulty in making a correct prediction.

We display qualitative results on unseen species in **Figure 9**. These segmentations vary widely in quality depending on the specific images, as seen in the first two rows, which display results for visually similar images. Therefore, validating the results for unseen species is even more important. This figure also displays plausible mistakes (b) and missing species-specific knowledge (b and c) from the multi-species model. However, it is the only model that provides acceptable ring estimates for unseen species.

### 3.3 Additional results

In the following subsections, we support our model design choices with experimental evidence.

#### 3.3.1 Radial regression model

We single out the design decisions for the radial regression model loss and show the step-wise improvements achieved by each component. All experiments are done with the same semantic segmentation model per species to exclude variances in the semantic prediction. We investigate the influence of the loss type and the target normalization, which maps the regression values from 0 to 255 to −1 to 1. **Table 3** shows clear improvements for each step, supporting our model design choices. Switching to the L1 loss, which is more robust against outliers, significantly improves performance with an average gain of 7 pts for mAR and 6 pts for PQ. For the DO, the performance difference is drastic, changing the MAE from 26.6 to 16.1, which is a reduction of nearly 40%. Adding the target normalization, formally described in Equation 3, displays similar improvements for EH and DO. For VM, however, this step is necessary to gain proper segmentation results, improving the performance by 34.6 pts for mAR, 20 pts for ARAND, and 27.5 pts for PQ. This resulted in an improvement from 37.1 to 7.96 for MAE and 22.2 to 2.66 for MedAE, which represents error reductions of 78% for MAE and 88% for MedAE. These results show the need for the L1 loss in combination with target normalization, which stabilizes the training and, therefore, results in the best performance for each species.

**Table 3:** Results of the ablation study for loss type.

	Method	mAR↑	ARAND↓	PQ↑	SQ↑	RQ↑	MAE↓	MedAE↓
EH	L2	0.746	0.101	0.788	0.865	0.911	11.9	4.09
	L1	0.777	0.089	0.810	0.878	0.923	10.2	3.17
	L1 target norm	**0.823**	**0.077**	**0.837**	**0.897**	**0.932**	**6.91**	**2.49**
DO	L2	0.579	0.193	0.661	0.799	0.827	26.6	15.2
	L1	0.686	0.142	0.747	0.843	0.886	16.1	7.84
	L1 target norm	**0.760**	**0.103**	**0.797**	**0.862**	**0.925**	**13.2**	**5.85**
VM	L2	0.410	0.300	0.446	0.728	0.614	52.4	38.6
	L1	0.480	0.260	0.520	0.759	0.685	37.1	22.2
	L1 target norm	**0.826**	**0.061**	**0.795**	**0.907**	**0.877**	**7.96**	**2.66**

This study shows the importance of the used L1 loss in combination with the target normalization and compares it to the commonly used L2 loss. Bold highlights the best performance. Arrows indicate values of higher performance.

#### 3.3.2 Uncertainty estimation

We investigate how well uncertainties are calibrated and determine if the additional uncertainty estimation deteriorates the overall performance. This is done for each species individually. We evaluate the uncertainty calibration with the previously described ENCE metric, which gives a good intuition of the uncertainty quality.

We report the ring segmentation metrics in **Table 4**. They show similar performance between the method with and without uncertainty calibration if we use the Laplace distribution for the NLL loss. We additionally investigate the performance with the Gaussian distribution that is commonly used by default for uncertainty estimation. Since NLL with a Gaussian distribution is related to an L2 loss, we can see some performance degradation, as shown in **Table 4**. Additionally, we see a large difference between the Gaussian and Laplacian NLL for the ENCE metric. On average, the ENCE metric is 97% lower for the Laplacian NLL, clearly showing a better calibration of the uncertainty.

**Table 4:** Results of the ablation study for uncertainty estimation.

	Method	mAR↑	ARAND↓	PQ↑	SQ↑	RQ↑	MAE↓	MedAE↓	ENCE↓
EH	L1	**0.823**	0.077	0.837	**0.897**	0.932	6.91	2.49	–
	NLL gauss	0.791	0.086	0.815	0.884	0.922	8.06	2.82	20.6
	NLL	**0.823**	**0.075**	**0.844**	**0.897**	**0.940**	**6.51**	**2.42**	**0.973**
DO	L1	**0.760**	**0.103**	**0.797**	**0.862**	**0.925**	13.2	5.85	–
	NLL gauss	0.739	0.113	0.783	0.852	0.918	13.5	6.06	329.
	NLL	0.755	0.107	0.792	0.861	0.920	**13.0**	**5.79**	**4.57**
VM	L1	**0.826**	**0.061**	0.795	0.907	0.877	7.96	2.66	–
	NLL gauss	0.818	0.065	**0.799**	0.904	**0.884**	8.25	2.85	35.1
	NLL	0.821	0.062	0.790	**0.910**	0.868	**7.42**	**2.45**	**0.528**

L1 loss represents the baseline results without uncertainty, NLL represents the method with uncertainty using a Laplacian noise assumption, and NLL gauss represents the comparison method using a Gaussian noise assumption. See Methods for an explanation of the different performance metrics. Arrows indicate values of higher performance. Bold highlights the best performance.

#### 3.3.3 Multi-species training

In this evaluation, we further investigate the results of our multi-species training and, specifically, the performance increase for EH and VM and the decrease for DO. By looking at the performance of the semantic segmentation model, we see a clear difference between the species, shown in **Table 5**. In each species, the boundary segmentation improves by roughly 1pt, however, we can see a drop in performance for the pith and background only for DO. The DO species contains samples with piths that were torn off during the thin-sectioning process, giving it the same visual appearance as the background. Additionally, some samples have no rings on one side, so there is a direct connection between the pith and the background. Both these cases make differentiating between the pith and the background more difficult. An example for both cases can be seen in **Figure 4**. This is especially true for the multi-species case, where these difficult cases are an even smaller percentage compared to the single-species case. These errors in pith prediction will propagate through multiple iterative steps, resulting in a decreased segmentation performance for DO. In the other species, the improved boundary prediction increases the overall performance even further.

**Table 5:** Results of multi-species training for the semantic segmentation model.

	Dataset	mIoU Pith↑	mIoU Bark↑	mIoU Rings↑	mIoU Boundary↑
EH	Single	0.888	0.983	0.981	0.463
	Multi	**0.900**	0.980	0.981	**0.470**
DO	Single	**0.880**	**0.974**	0.965	0.288
	Multi	0.790	0.964	0.967	**0.298**
VM	Single	0.943	0.997	0.983	0.419
	Multi	0.942	0.997	0.985	**0.430**

The values are marked bold if the difference is larger than 0.5%. Arrows indicate values of higher performance.

### 3.4 Limitations and further work

We observe problems with improper pith predictions if the pith of the sample is broken or looks visually similar to the background, or for species unseen during training. These improperly segmented piths lead to follow-up errors due to the iterative nature of our method. The same difficulties can be seen in the INBD method. Additionally, not detecting a pith automatically prevents the model from being used on unseen species without human intervention. Since pith prediction is only necessary for the first step, any pith prediction method can be directly integrated into the existing method, which makes it an ideal area for further development.

Another problem is when the rings are very narrow on one side of the pith. For these rings, properly detecting the correct boundary becomes nearly impossible. Even experts struggle in these regions, which makes the annotations less reliable, increasing the difficulty even further. These less reliable labels directly influence the uncertainty estimation and make evaluation even more challenging. Further research could investigate increasing the robustness of the uncertainty, incorporating the uncertainty directly in the iterative process, and adding uncertainty prediction to the pith prediction.

## 4 Conclusion

In this study, we aimed to develop a deep learning-based model for ring boundary detection in anatomical images using the existing INBD model as a starting point and benchmark. Using a regression approach shows clear performance improvements in combination with the possibility of further enhancing usability through uncertainty estimation. The indication of uncertain rings and ring segments is particularly important for downstream applications as it can guide human users to target specific rings for editing, thus substantially reducing operator time. Additionally, uncertainties could be used to automatically select the most certain portion of the ring for ring width estimation or exclude the most uncertain ring segments. Moreover, we showed that training our model on multiple species can double the segmentation performance as measured by certain quality metrics for unseen species. This is facilitated by our iterative unrolling training procedure, which allows our model to be trained on larger datasets. However, the performance drop between unseen and seen species clearly shows the need for larger and more diverse datasets to train a model that achieves human-level segmentation performance on unseen species. Our work lays the methodological foundation to use such a large and diverse dataset. This methodological foundation will help to tackle the related problem of linear tree ring structures and conifer anatomies, bringing us one step closer to an AI-based ROXAS.

## Data Availability

The raw data supporting the conclusions of this article will be made available by the authors, without undue reservation. Additionally, the source code of our method will be open-sourced after publication under https://marckatzenmaier.github.io/TowardsRoxasAI-ring-segmentation/.
