# Supplementary material for: Towards ROXAS AI: automatic multi-species ring boundaries segmentation as regression in anatomical images
Source: Front Plant Sci. 2025 May 6;16:1516635. doi: 10.3389/fpls.2025.1516635 (PMC12090873; doi:10.3389/fpls.2025.1516635)
Supplement: Supplementary file 1 [file DataSheet1.pdf]

Supplementary Material

1 ADDITIONAL RESULTS FOR DIFFICULT TO EVALUATE SAMPLES

In fig. S1 we show additional results with especially difficult to predict samples. For the sample of species EH it is even difficult to assign proper labels. For the DO sample, our method shows good results for most rings however it struggles to understand the broken side of the first ring. For the VM sample, it is debatable if our result or the annotation which highlights the crack as background is more useful for further processing.

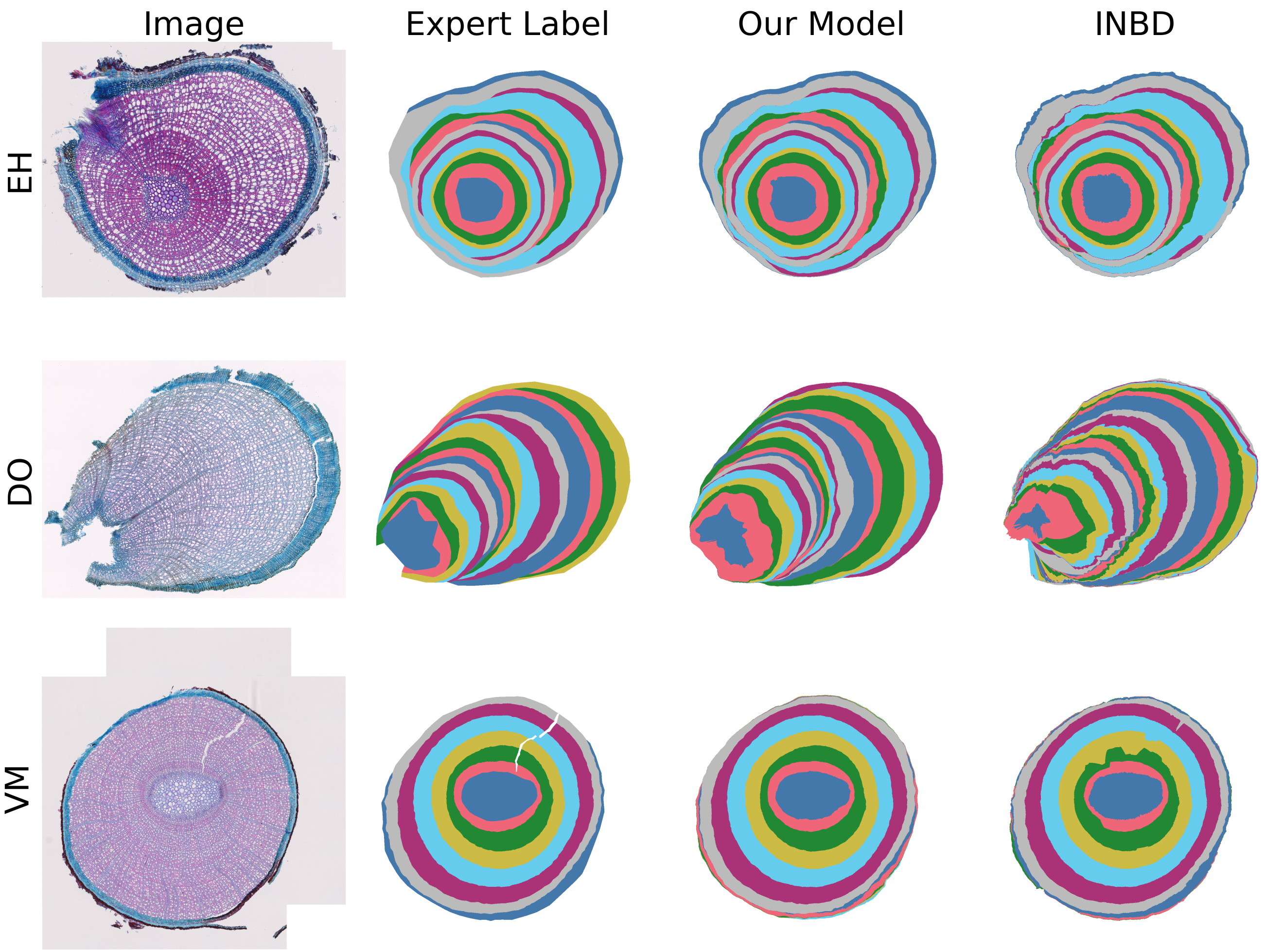

Figure S1. Additional visual results for difficult sample from each species. The EH sample has a large protrusion. The DO sample has no border between pith and background. Additionally, the rings are only on one side of the pith. The VM sample has a noticeable crack through most rings

2 ERROR FOR DIFFERENT RING NUMBERS

In this section, we further investigate the error for different ring numbers. Figure S2 displays a larger mean and median absolute error for the first ring. This can be explained by the irregularisations of piths and therefore difficulties to detect them properly. The immediately following rings show the lowest error before it slightly increases with higher numbers. By inspecting the results visually we could not confirm this trend. However, we noticed thinner and more wedging rings for higher ring numbers, especially if the pith is off-center. In addition to our observation, the number of rings decreases with higher ring numbers. Especially for the largest numbers where the performance decreases most noticeably the low ring count does not allow for a meaningful conclusion. Low ring counts also mean a reduced number of training samples which could further impact the segmentation performance.

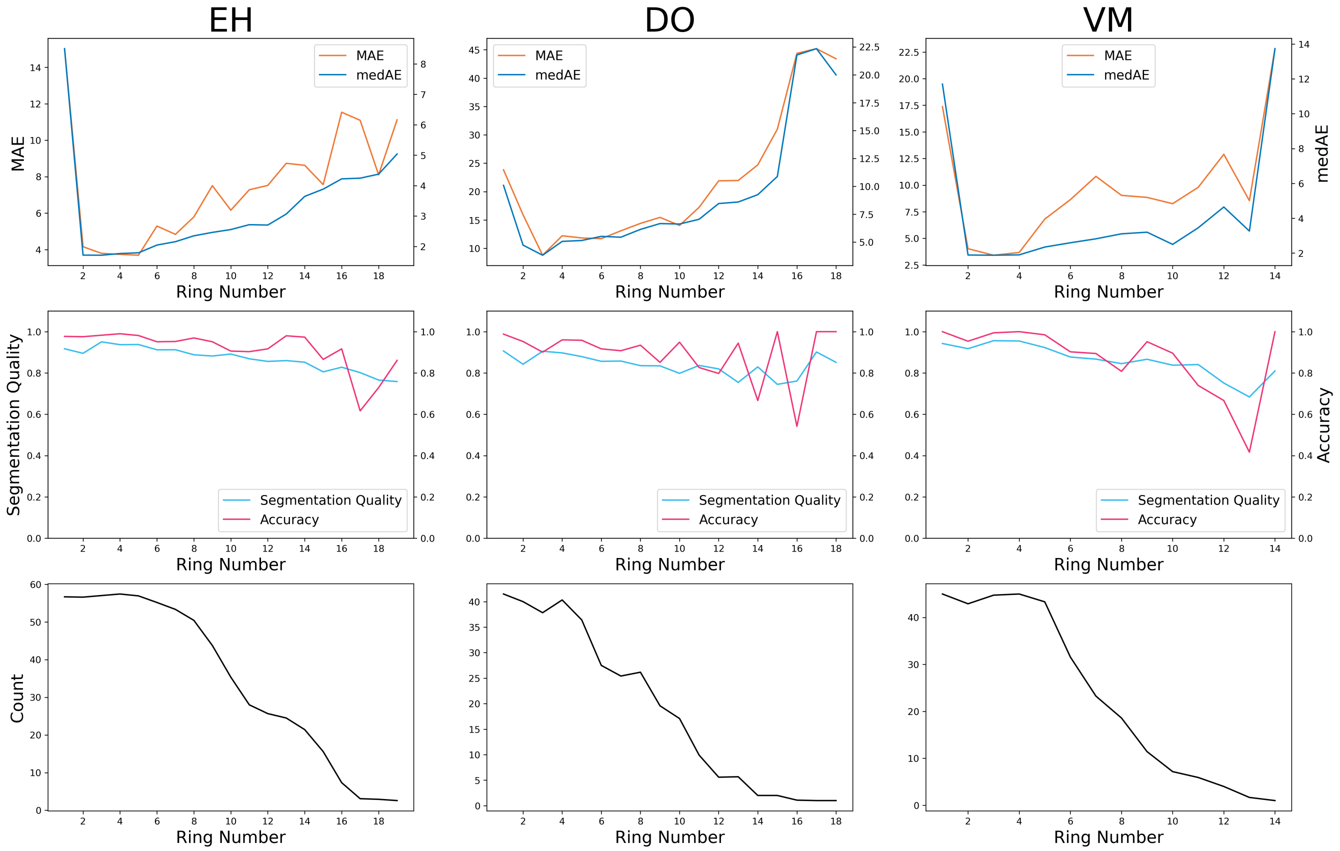

Figure S2. Visualisation of the metrics over the ring number. Top row shows the mean absolute error (MAE) and median absolute error (medAE), second row shows the segmentation quality and the accuracy. We use the accuracy since the recognition quality can not be calculated for a single ring due to the missing false positive prediction. The third row visualizes how many rings are there for each ring number.

3 INTERPOLATION USED IN INBD-R

In table S1 we display which interpolation is used for the downsampling before the semantic segmentation model and for the polar grid interpolation.

Table S1. Interpolation methods used. Pre semantic segmentation refers to the downsampling before the semantic segmentation and polar grid interpolation refers to the interpolation method used for polar image creation. Inter area > 0 is a custom interpolation by first applying inter area interpolation followed by setting all values lager than 0 to one. This assures connected and visible boundary lines.

	pre semantic segmentation	polar grid interpolation
image	bilinear	bilinear
label mask	nearest neighbour	nearest neighbour
boundary mask	inter area > 0	-
semantic segmentation features	-	bilinear
